# Polymorphism of the *FSHB* Gene Is Associated with Endometrial Hyperplasia

**DOI:** 10.3390/life16050782

**Published:** 2026-05-07

**Authors:** Vladimir Churnosov, Maria Churnosova, Evgeny Reshetnikov, Inna Aristova, Kirill Tsoy, Inna Sorokina, Alexey Polonikov, Maria Solodilova, Mikhail Churnosov, Irina Ponomarenko

**Affiliations:** 1 Department of Medical Biological Disciplines, Belgorod State National Research University, 308015 Belgorod, Russia; 958561@bsuedu.ru (V.C.); churnosovamary@gmail.com (M.C.); reshetnikov@bsuedu.ru (E.R.); aristova@bsuedu.ru (I.A.); tsoy@bsuedu.ru (K.T.); sorokina@bsuedu.ru (I.S.); polonikov@rambler.ru (A.P.); solodilovama@kursksmu.net (M.S.); ponomarenko_i@bsuedu.ru (I.P.); 2 Department of Biology, Medical Genetics and Ecology, Kursk State Medical University, 305041 Kursk, Russia; 3 Research Institute for Genetic and Molecular Epidemiology, Kursk State Medical University, 305041 Kursk, Russia

**Keywords:** sex hormones, endometrial hyperplasia, SNP, association

## Abstract

The work was performed to assess the relationship of single-nucleotide polymorphisms (SNPs), which determine the concentration of sex hormones (confirmed in previously performed genome-wide studies (GWASs)), with the risk of endometrial hyperplasia (EH). The objects of the study were 1493 women, of which 520 individuals had EH; the control group consisted of 973 women. Nine SNPs that were GWAS-associated with the level of sex hormones were investigated. The correlations of SNPs that determine the level of sex hormones with EH risk were found: minor polymorphic variants rs11031002 (allele A: OR = 0.45–0.50) and rs11031005 (allele C: OR = 0.05–0.53) of the *FSHB* gene were associated with a low risk of developing the disease, and the TT*rs11031002-rs11031005 *FSHB* haplotype, at a level of statistical significance (*p* = 1 × 10^−11^) exceeding the GWAS “standard”, increases the EH risk by more than 2.5 times (OR = 2.84). The 16 multilevel SNP interaction exploratory models of nine considered loci were EH-associated (*p*_adj-perm_ < 0.001). Two loci, T>A rs11031002 and T>C rs11031005 *FSHB*, play a fundamental role in these models (100% and 75% of models, respectively), and two more loci, C>G rs112295236 *SLC22A10* and C>A rs117585797 *ANO2*, are part of more than 30% of all models. Sex hormone-level genetic determinants are involved in numerous EH-significant hormone-mediated molecular pathways (regulation of gene transcription, processes of embryogenesis and development, regulation of metabolism, differentiation and maturation of the epithelium, TGFβ pathway, fat cell differentiation, etc.). In conclusion, for the first time, it was found that the genetic polymorphisms that determine an organism’s sex hormone levels are associated with EH.

## 1. Introduction

Endometrial hyperplasia (EH) is a gynecological disease affecting the uterus endometrium, in which a proliferation of glands and an increase in the glandular–stromal ratio occur [1]. The prevalence of EH in different populations varies widely—from 37 to 132 per 100,000 female-years [2,3,4]. The incidence of EH among women increases significantly with age and can reach, according to various estimates, levels of 270 cases per 100,000 female-years in individuals aged 46–49 years [3,4] and 386 cases per 100,000 female-years in women aged 50–54 years [2]. In premenopausal women with infertility, EH occurs in 0.9–3.0%, and in women with abnormal uterine bleeding, the frequency of EH is significantly higher and varies from 3.4% to 26.5% [4].

EH is characterized by a long and recurrent course, often manifested by abnormal uterine bleeding, which significantly reduces the quality of life of patients, as well as the presence of an increased risk of malignant transformation of the endometrium [4,5,6]. The risk of endometrial cancer in women with EH varies from 1% in simple hyperplasia without atypia to 29% in complex atypical hyperplasia [5]. The literature indicates that, on average, 6 years after the initial diagnosis of atypical EH, endometrial cancer is detected [6]. It is noted that upon repeated “independent” morphological examination of endometrial samples with atypical EH, cases of malignancy foci are detected in 29.1%, which gives reason to consider atypical EH as the equivalent of “early” endometrial cancer [6].

The development of EH is the result of the influence of various risk factors (genetic, woman age, obesity, diabetes mellitus, chronic anovulation, earlier menarche/late menopause, prolonged perimenopause, postmenopausal status, long-term estrogen therapy, etc. [3,4,5,6,7]), with the involvement of hormone-dependent and hormone-independent processes regulating proliferation, angiogenesis, apoptosis of endometrial cells, various metabolic and immune disorders, etc. [4,5,6,7,8,9], the role of which in the formation of the disease is not always unambiguous; this causes numerous discussions among specialists and requires further research.

The role of genetic factors in the formation of EH has so far been studied extremely poorly: there are no estimates of the heritability of the disease and the contribution of genetic factors to its occurrence; genome-wide studies (GWASs) of EH have not yet been conducted, and only a few data were obtained in studies of polymorphism associations of individual groups of candidate genes (cytochromes, factors growth and tumor necrosis, interleukins and chemokines, estrogen receptors, apoptosis, age at menarche genes, etc.) with the risk of developing the disease [10,11,12,13,14,15], which is certainly insufficient and requires the significant pursuit of further genetic and epidemiological studies in this area.

The literature data clearly indicate the important role of sex hormones and disorders in their concentrations/ratios in EH pathophysiology: the development of hyperestrogenism (absolute or relative) with a lack of progesterone effects, an imbalance in the ratio of follicle-stimulating (FSH)/luteinizing hormones (LH), leading to the appearance of anovulatory cycles, androgenic effects on the endometrium, etc. [5,6,7,8,9]. Numerous GWASs conducted to date have revealed a number of single-nucleotide polymorphisms (SNPs) that determine the level of sex hormones (estradiol, progesterone, testosterone, sex hormone-binding globulin (SHBG), LH and FSH, dehydroepiandrosterone sulfate (DHEAS), etc.) and their metabolites in an organism [16,17,18,19,20,21,22,23,24,25,26]. It can be assumed with high probability that polymorphisms that determine the concentrations of sex hormones and their metabolites in the organism may be involved in EH pathogenesis. This work is devoted to solving this scientific problem. It should be emphasized that, to date, no such studies have been conducted in the world.

## 2. Materials and Methods

### 2.1. Study Subjects

The objects of the study were 1493 women, who gave their consent (in writing) to participate in this investigation (the design of the study was approved by the regional Ethics Commission during its planning), of whom 520 individuals had EH; the control group consisted of 973 women. All the study participants were Russians born/living in Central Russia, without close family ties [27]. Sampling and verification of the EH diagnosis were performed by certified gynecologists of the perinatal center of the Regional Clinical Hospital during 2008–2013. The group of patients included women with hyperplasia without atypia [28], with mandatory morphological confirmation of the diagnosis (all patients underwent morphological examination of endometrial samples obtained by hysteroscopy/curettage) [15]. The control group consisted of women who had no (anamnestic and clinical/ultrasound examination) symptoms of pelvic organ diseases. The reason for exclusion from the formed sample was the presence of oncological pathology or severe diseases of the immune system and vital organs in the examined person [29,30,31]. According to the materials in Table 1, which provide detailed characteristics of the study participants (EH; control) (Table 1 contains information provided by us in an earlier genetic study of EH, in the same sample of patients and in a similar control cohort [15]), the EH group differs from the control group, with a higher weight/BMI (*p* < 0.001), higher proportion of overweight/obese individuals (*p* < 0.001), increased family history of benign proliferative diseases of the uterus (*p* < 0.001), increased history of infertility (*p* < 0.001), higher rates of chronic endometritis (*p* < 0.001), fewer births (*p* < 0.001) and a higher number of induced abortions (*p* < 0.001), which served as the basis for using these factors as confounders in assessing the relationship between SNP and EH.

### 2.2. SNPs Linked with Sex Hormones, Laboratory Examination

DNA samples of EH/control, stored in the biobank of the Belgorod State University (department of biomedical disciplines), were used for this study (samples were obtained earlier during genetic research of EH in 2008–2013 [15]. Nine SNPs, including T>C rs148982377 *ZNF789*, G>T rs34670419 *ZKSCAN5*, T>A rs11031002 *FSHB*, T>C rs11031005 *FSHB*, C>G rs112295236 *SLC22A10*, C>A rs117585797 *ANO2*, A>C rs117145500 *CHD9*, C>T rs727428 *SHBG*, and C>T rs1641549 *TP53*, were investigated. These loci are functionally significant (information obtained from HaploReg [32], Table S1) and GWAS-associated with the level of sex hormones (Table S2), such as estradiol [18,22], testosterone [total/bioavailable] and its metabolites [19,20,22,23,24,25], DHEAS [17,18], progesterone [18], SHBG [16,18,20,26], LH [18], FSH/FSHB [18,21], the free androgen index (FAI) [18,24], and the cortisol/DHEAS ratio [17]. To establish the genotypes of patients/controls according to the nine studied SNPs, a standard TaqMan PCR genotyping procedure (CFX96 [Bio-Rad Laboratories, USA] system) was used [33], with mandatory quality control of the experimental data obtained by additional re-genotyping of a certain number (about 5%) of the studied DNA samples from both patients and controls [34,35].

### 2.3. Association Analysis

The search for associations between individual SNPs/their haplotypes was performed based on the computation of OR with 95%CI indicators widely used in genetic research in the gPlink program (v. 1.07) [36] (calculations were performed in four genetic models—additive; dominant; recessive; allelic [37]), taking into account confounders (their list is given above) and confirmation of the identified associations through permutations [38]. P_adj-perm_ values equal to or less than 0.0125 (0.05/4, Bonferroni correction was introduced for the number of genetic models considered [39,40]) and 0.05 were considered statistically significant when evaluating associations of individual SNPs and their haplotypes, respectively. For the established EH-SNP associations, a power assessment was performed in the Quanto resource [41].

The MB-MDR program (implemented in the R package) [42] was used for exploratory modeling of intergenic/interlocus EH-significant interactions, and the necessary above-mentioned confounders were taken into account. To confirm the permutation method [38] of EH-significant models of interlocus interactions, models whose statistical significance level was not lower than the following values were selected (the Bonferroni correction was introduced, taking into account the number of possible combinations of nine SNPs for models of different levels) [43,44]: 2-level models—*p*_Bonferroni_ < 1.38 × 10^−3^ [0.05/36]; 3-level—*p*_Bonferroni_ < 5.95 × 10^−4^ [0.05/84]; 4-level—*p*_Bonferroni_ < 3.97 × 10^−4^ [0.05/126]. The interlocus interaction model’s *p*_adj-perm_ parameter < 0.001 was taken into account as statistically relevant. In order to visually represent the EH-significant interlocus interactions, a graph was built in the MDR program [45].

### 2.4. Study of How SNPs–Genes–Proteins Predict Functions

At the final stage of study, a multilateral assessment of the functionality of EH-related loci and strongly linked variants (r^2^ ≥ 0.8) [46] was carried out (in silico methodology has been applied [40] and a number of the following bioinformatic online resources were used for this purpose [47,48]: HaploReg (accessed on 15 April 2024) [32]; PolyPhen2 (accessed on 18 April 2024) [49], GTExportal (accessed on 20 April 2024) [50]; SIFT (accessed on 25 April 2024) [51], Gene Ontology (accessed on 28 April 2024) [52]; STRING (accessed on 12 May 2024) [53]).

## 3. Results

The genotype/allele frequencies of the examined loci in EH/control cohorts matched HWE (*p*_Bonferroni_ > 0.006 (0.05/9) (the data is presented in Table S3)).

Our search for the associations of SNPs with the risk of EH revealed a connection in the disease with two polymorphisms of the *FSHB* gene—T>A rs11031002 and T>C rs11031005—both independently (Table 2) and as part of haplotypes (Table 3). It has been established that allelic variants A*rs11031002 and C*rs11031005 were protective factors in the occurrence of EH. Polymorphism T>C rs11031005 *FSHB* was EH-associated according to all four considered genetic models: allelic [OR = 0.52; 95%CI = 0.40–0.68; *p* = 1 × 10^−6^; *p*_adj-perm_ = 3 × 10^−6^], additive [OR = 0.51; 95%CI = 0.38–0.69; *p* = 8 × 10^−6^; *p*_adj-perm_ = 0.00002; power = 99.84%), dominant [OR = 0.53; 95%CI = 0.39–0.73; *p* = 0.00007; *p*_adj-perm_ = 0.00009; power = 99.22%], and recessive [OR = 0.05; 95%CI = 0.01–0.39; *p* = 0.005; *p*_adj-perm_ = 0.008; power = 86.71%]. The locus T>A rs11031002 *FSHB* was EH-correlated within the framework of three genetic models: allelic [OR = 0.50; 95%CI = 0.38–0.66; *p* = 5 × 10^−7^; *p*_adj-perm_ = 1 × 10^−6^], additive [OR = 0.45; 95%CI = 0.33–0.61; *p* = 4 × 10^−7^; *p*_adj-perm_ = 1 × 10^−6^; power = 99.99%], and dominant [OR = 0.43; 95%CI = 0.31–0.59; *p* = 3 × 10^−7^, *p*_adj-perm_ = 1 × 10^−6^; power = 99.99%] (Table 2).

It was found that the most common TT*rs11031002-rs11031005 haplotype, both among patients with EH (91.92%) and in the control (84.16%), has the most statistically pronounced associations with EH risk [*p* = 1 × 10^−11^; *p*_adj-perm_ = 1 × 10^−6^]. It is important to note that the level of statistical significance of the association of this haplotype with EH significantly exceeds (by more than three orders of magnitude) the similar indicator adopted as a “threshold” in genome-wide studies (*p* = 5 × 10^−8^) The presence of this haplotype in a woman’s genotype increases her EH risk more than 2.5 times [OR = 2.84]. The appearance of one or two minor SNP alleles, rs11031002 (allele A) and rs11031005 (allele C), in the haplotype leads to a significant reduction in the risk of disease, and any combinations involving these alleles in the haplotype already have a protective value for EH formation [OR < 1] (Table 3).

Based on the exploratory analysis of interlocus interactions, the involvement of all nine analyzed loci in EH susceptibility was revealed (Table 4). The SNP data, interacting with each other within the framework of the 16 models (*p*_adj-perm_ < 0.001) of different levels (six models—four levels and five models each—two and three levels), determine the risk of developing EH. It should be emphasized that the “basic” real statistical significance of these models (prior to permutation testing) significantly exceeds the “threshold” values set by us, taking into account the Bonferroni correction (*p*_Bonferroni_) for the maximum possible number of combinations of the nine analyzed loci at different levels of their interlocus interactions: two-level models—*p*_Bonferroni_ < 1.38 × 10^−3^, with a real value of *p* < 7.57 × 10^−7^; three-level model—*p*_Bonferroni_ < 5.95 × 10^−4^, with a real value of *p* < 2.52 × 10^−10^; four-level model—*p*_Bonferroni_ < 3.97 × 10^−4^, with a real value of *p* < 2.05 × 10^−10^. This indicates the high degree of reliability of the results obtained.

**Figure 1:**
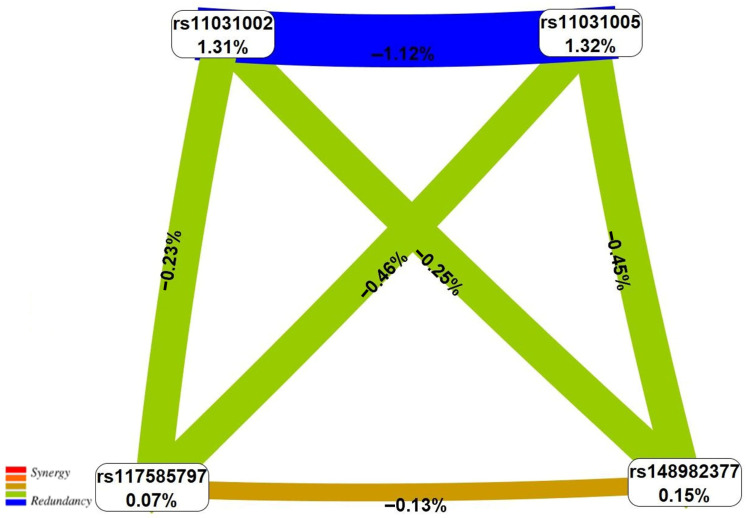
Graph of the most significant four-locus SNP × SNP interaction of sex hormone genes (rs11031002 *FSHB* × rs117585797 *ANO2* × rs11031005 *FSHB* × rs148982377 *ZNF789*, Wald statistics—45.99, *p*_perm_ < 0.001) associated with EH (obtained with MDR method). Positive values of entropy indicate synergistic interactions, while the negative values indicate redundancy. The brown color denotes an independent effect, and green and blue colors denote moderate and strong antagonism.

According to the information in Table 4, all 16 models include polymorphism T>A rs11031002 *FSHB*, and SNP T>C rs11031005 *FSHB* is an integral part of 12 models (75.00%). Two loci—C>G rs112295236 *SLC22A10* and C>A rs117585797 *ANO2*—affect the EH risk within five models each (31.25%) (Table 4). The two-locus interaction rs11031002 *FSHB* × rs11031005 *FSHB* is the basis of all five three-locus and all six four-locus models. The most “crucial” risk effect for EH was the four-locus model (rs11031002 *FSHB* × rs117585797 *ANO* × rs11031005 *FSHB* × rs148982377 *ZNF789*), characterized by the highest Wald statistic index—45.99 (Table S4 and Figure 1).

Importantly, the association of nine different genotype combinations with EH has a genome-wide level (and higher) of statistical significance: rs11031002 × TT × rs117585797 × CC × rs11031005 × TT (*beta* = 0.92, *p* = 2 × 10^−10^), rs11031002 × TA × rs11031005 × TT (*beta* = −3.00, *p* = 2 × 10^−9^), rs11031002 × TA × rs117585797 × CC × rs11031005 × TT (*beta* = −3.26, *p* = 3 × 10^−9^), rs11031002 × TT × rs112295236 × CC × rs11031005 × TT (*beta* = −3.56, *p* = 9 × 10^−9^), rs11031002 × TA × rs117585797 × CC × rs112295236 × CC × rs11031005 × TT (*beta* = −3.56, *p* = 9 × 10^−9^), rs11031002 × TA × rs117585797 × CC × rs11031005 × TT × rs148982377 × TT (*beta* = −3.10, *p* = 2 × 10^−8^), rs11031002 × TA × rs11031005 × TT × rs148982377 × TT (*beta* = −2.84, *p* = 2 × 10^−8^), rs11031002 × TT × rs11031005 × TT (*beta* = 1.03, *p* = 3 × 10^−8^), and rs11031002 × TA × rs112295236 × CC × rs11031005 × TT × rs148982377 × TT (*beta* = −3.31, *p* = 5 × 10^−8^) (Table S5).

**Figure 2:**
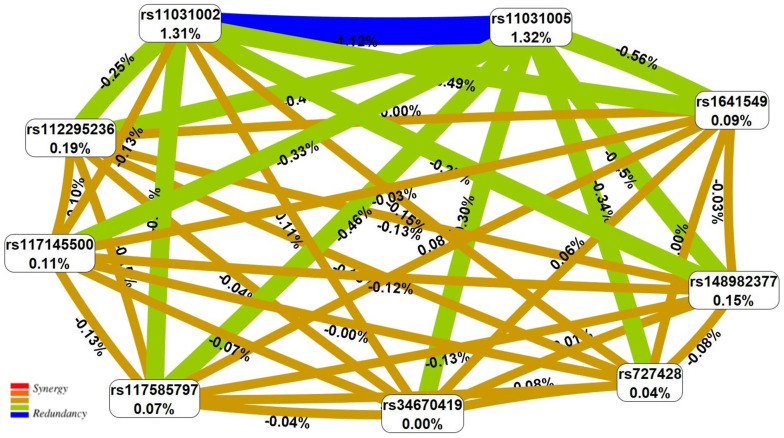
The entropy graph of the SNP × SNP interactions with EH based on the MDR analysis. Positive values of entropy indicate synergistic interactions, while the negative values indicate redundancy. The brown color denotes an independent effect, and green and blue colors denote moderate and strong antagonism.

The results of the performed visualization of the interlocus interactions determining EH risk, both within the framework of the most significant four-locus model (rs11031002 *FSHB* × rs117585797 *ANO2* × rs11031005 *FSHB*× s148982377 *ZNF789*) and when considering all nine SNPs significant for the occurrence of the disease, are shown in Figure 1 and Figure 2. Within the framework of the most significant EH-associated four-locus model, attention is drawn to the pronounced epistatic interaction of the antagonistic orientation of two SNPs of the *FSHB* gene—rs11031002 and rs11031005. The potential contribution of this two-locus interaction to EH susceptibility reaches 1.12% and is comparable to the main effects of these loci—1.31% and 1.32%, respectively (Figure 1 and Figure 2). When considering the interlocus interactions of all nine EH-associated SNPs, the overall “picture” did not change—rs11031002 and rs11031005 of the *FSHB* gene have a dominant influence on the development of the disease among all EH-significant loci, showing both pronounced independent effects and epistatic interactions most important for the disorder, the contribution of which to the entropy of the trait (EH development risk) significantly exceeds both the effects of two-focus interactions (by more than 2 times) and the main effects (by more than 6 times) of other SNPs (Figure 2).

### 3.1. Alleged Functionality of EH-Significant Loci

#### 3.1.1. Missense Mutation of Genes Exons

The materials presented in the PolyPhen and SIFT databases show that only one locus of the 90 polymorphisms considered (rs1042522 *TP53* is strongly linked [r^2^ = 0.88] with the EH-causal SNP C>T rs1641549 *TP53*) is a missense mutation, with a presumed predictive potential that is “benign”/“tolerated” for amino acid substitution P72R in the TP53 protein (Score_PolyPhen_ = 0.0083/Score_SIFT_ = 0.493).

#### 3.1.2. Epigenetic Modifications

Among the 90 EH-involved SNPs considered, 54 loci (60.00%) were located in genes [one SNP (1.11%) in the exon of the *TP53* gene, leading to the replacement of the amino acid P72R in the TP53 protein; 53 SNPs (58.89%) in the introns of the genes *ANO2*, *ZNF789*, *SLC22A24*, *SHBG*, *SLC22A25*, *TP53*], with one locus (1.11%) in the 5′-UTR of *TP53*, 30 SNPs (33.33%) in the 5′-UTR of *CHD9*, *FSHB*, *SLC22A25*, and *SLC22A24*, two loci (2.22%) in the 3′-UTR of *ZKSCAN5*, and 10 SNPs (11.11%) in the 3′-UTR of *RP11-467J12.4*, *SHBG*, and *SLC22A25* (Table S5). Six loci (6.67%) were located in conservative regions of *FSHB*, *SLC22A10*, and *SLC22A25*; eight SNPs (8.89%) were in promotors of *SHBG*, *SLC22A10*, and *SLC22A25*, *TP53*; 17 SNPs (18.89%) were in enhancers of *SHBG*, *SLC22A10*, *SLC22A25*, *TP53*, *SLC22A24*, and *ZNF789*; 12 SNPs (13.33%) were in areas of “open” chromatin (DNase-hypersensitive sites) of *SHBG*, *SLC22A10*, *SLC22A25*, *TP53*, *SLC22A24*, and *ZNF789*; four SNPs (4.44%) were in sites of *SHBG*, *SLC22A24*, *SLC22A10*, and *SLC22A25*, interacting with 15 regulatory proteins (FOXA1, SP1, FOXA2, CFOS, P300, GATA2, RAD21, CTCF, SMC3, HDAC2, TCF4, MAFF, CEBPB, RXRA, MAFK); and 81 SNPs (90.00%) were in regions of *CHD9*, *ANO2*, *ZKSCAN5*, *RP11-467J12.4*, *SHBG*, *FSHB*, *SLC22A24*, *SLC22A10*, *SLC22A25*, *TP53*, and *ZNF789*, interacting with transcription factors (TFs). In total, we have registered various epigenetic effects of 90 EH-linked SNPs on 11 genes [*CHD9*, *ANO2*, *RP11-467J12.4*, *SHBG*, *FSHB*, *SLC22A24*, *SLC22A10*, *SLC22A25*, *ZNF789*, *TP53*, *ZKSCAN5*] (Table S5).

Interestingly, two EH-significant polymorphisms, T>A rs11031002 and T>C rs11031005 *FSHB* (independently associated with the disease), were located in the regions of “DNA-TF” interaction with four (HDAC2, Pou2f2, Pou6f, Zfp105) and two (Otx2, Zfp281) TFs, respectively (Table S2). At the same time, allelic variants of these polymorphisms that were protective for EH (A*rs11031002 and C*rs11031005) increase the “sensitivity” of DNA to the effects of TFs HDAC2, Pou2f2, and Zfp105 and reduce the affinity of DNA to the action of TFs Otx2 and Pou6f1. In addition, T>A rs11031002 was located in the enhancer position of *FSHB* in the ovaries.

#### 3.1.3. Regulation of Gene Expression (eQTL)

It was revealed that minor alleles of two EH-causal polymorphisms, T>A rs11031002 and T>C rs11031005 *FSHB*, were associated with higher transcription of the *ARL14EP* gene in more than ten different organs (thyroid gland, adipose tissue, etc.) (Table S6). Overall, among the 90 EH-related SNPs, 73 loci have eQTL influences (81.11%; seven EH-causal loci and 66 LD SNPs) with respect to 23 different genes (*ATL3*, *ARL14EP*, *ATP1B2*, *CYP3A7*, *CHRNB1*, *GS1-259H13.2*, *EFNB3*, *SLC22A10*, *FGF11*, *FXR2*, *EIF4A1*, *KDM6B*, *SAT2*, *OR2AE1*, *PTCD1*, *SENP3*, *SHBG*, *SLC22A9*, *TNFSF12*, *TNFSF13*, *SOX15*, *ZKSCAN5*, *TRIM4*) (Tables S6 and S7). It is important to note the effect on gene expression of the loci under consideration in organs important for EH pathophysiology, such as the brain [basal ganglia (*ATP1B2*, *SOX15*), pituitary gland (*SHBG*)], thyroid gland (*ARL14EP*, *GS1-259H13.2*, *SHBG*, *EFNB3*), adrenal glands (CYP3A7), skeletal muscles (*CHRNB1*, *FGF11*, *SAT2*), adipose (*CYP3A7*, *ARL14EP*, *EFNB3*), mammary gland (*TRIM4*, *ARL14EP*, *EFNB3*), and blood (*TNFSF12*, *ZKSCAN5*, *CHRNB1*, *TNFSF13*, *FXR2*).

#### 3.1.4. Regulation of Gene Alternative Splicing (sQTL)

Three EH-associated loci (T>C rs148982377 *ZNF789*, G>T rs34670419 *ZKSCAN5*, C>T rs727428 *SHBG*) and five strongly linked loci have been involved in the sQTL regulation of seven genes (*GPC2*, *AC113189.5*, *FGF11*, *SAT2*, *FXR2*, *ZBTB4*, *TNFSF13*) (Tables S8 and S9). It is necessary to point out the sQTL effects of the above-mentioned SNPs in organs associated with EH pathogenesis, including the brain [the black substance (*GPC2*)], thyroid gland (*SAT2*, *FXR2*), skeletal muscles (*SAT2*, *AC113189.5*, *FXR2*), adipose tissue (*SAT2*, *AC113189.5*, *FXR2*), breast (*SAT2*), and blood (*SAT2*, *FXR2*, *TNFSF13*).

#### 3.1.5. Protein Interactions and Their Biological Pathways

**Figure 3:**
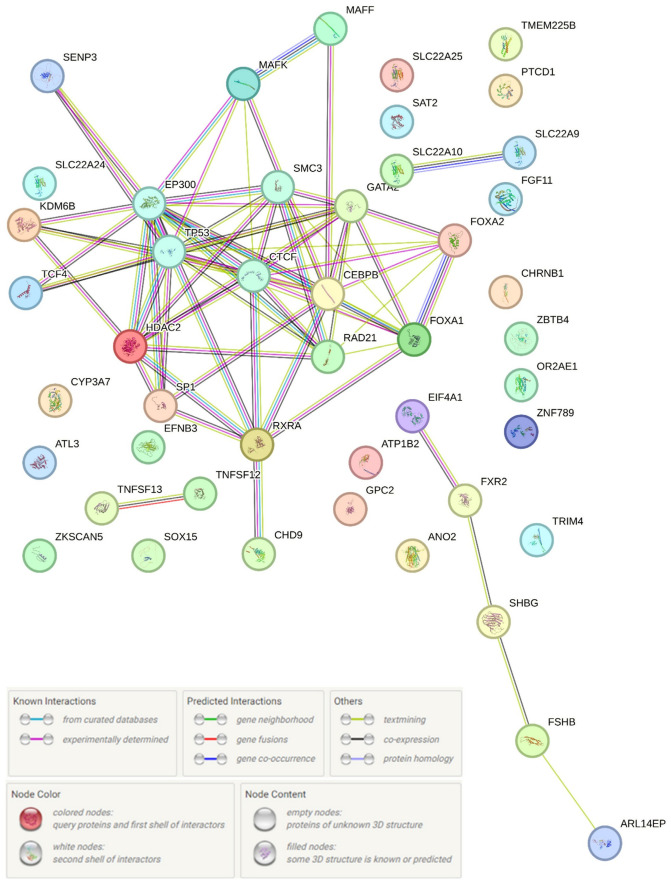
Protein interaction network related to the development of EH (STRING data, https://string-db.org/).

Using the STRING program, we evaluated the interaction of proteins encoded by 34 genes functionally associated with 90 EH-associated loci (*RP11-467J12.4*, *CHD9*, *AC113189.5*, *SLC22A9*, *ARL14EP*, *SOX15*, *ANO2*, *ATL3*, *CHRNB1*, *SLC22A25*, *ATP1B2*, *CYP3A7*, *FGF11*, *FXR2*, *EIF4A1*, *FSHB*, *EFNB3*, *GS1-259H13.2*, *OR2AE1*, *SAT2*, *SHBG*, *SLC22A24*, *LC22A10*, *TNFSF12*, *SENP3*, *TNFSF13*, *TP53*, *PTCD1*, *GPC2*, *TRIM4*, *ZBTB4*, *KDM6B*, *ZNF789*) and 15 regulatory proteins whose interaction with DNA is influenced by the SNPs we studied (FOXA1, SP1, FOXA2, CFOS, P300, GATA2, RAD21, CTCF, SMC3, HDAC2, TCF4, MAFF, CEBPB, RXRA, MAFK). Thus, we studied the interactions of 49 different proteins involved in EH pathophysiology according to our in silico data. The network of EH-associated protein interactions obtained as a result of this analysis is presented in Figure 3. As can be seen from the data presented in Figure 3, the interactions of regulatory proteins EP300 (12 interactions), CEBPB (11 interactions), and CTCF (11 interactions) are of key importance in EH-associated protein interactions. The most expressed paired protein interactions (score ≥ 0.99) were demonstrated by the regulatory proteins CTCF-RAD21, EP300-TP53, EP300-SP1, RAD21-SMC3, CEBPB-EP300, SP1-TP53, and HDAC2-TP53. Paired interactions such as FOXO1-FOXA2, CTCF-EP300, and EP300-SP1 were characterized by co-expression (co-expression score > 0.200), with the RAD21-SMC3 interaction having the most substantial value (co-expression score = 0.526).

Protein interactions associated with the formation of EH are mainly involved in the regulation of gene transcription, including the regulation of transcription by RNA polymerase II (GO: 000635720; *p* = 0.0067) (FOXA1, KDM6B, EP300, TP53, RAD21, CEBPB, ZBTB4, SP1, ZNF789, MAFK, GATA2, SOX15, ZKSCAN5, TCF4, FOXA2, FSHB, RXRA, HDAC2, MAFF, CTCF), chromatin organization (GO: 0006325; *p* = 0.0169) (FOXA1, KDM6B, EP300, TP53, SOX15, CHD9, FOXA2, HDAC2, CTCF), histone acetyltransferase binding (GO: 0035035; *p* = 0.0081) (TP53, CEBPB, SP1) and histone deacetylase binding (GO: 0042826; *p* = 0.0435) (TP53, CEBPB, SP1, HDAC2). RORA activates gene expression (HSA-1368082; *p* = 0.0064) (EP300, CHD9, RXRA), estrogen-dependent gene expression (HSA-9018519; *p* = 0.0064) (FOXA1, EP300, RAD21, SP1, SMC3), the transcriptional regulation of white adipocyte differentiation (HSA-381340; *p* = 0.0112) (EP300, CEBPB, CHD9, RXRA), cohesin loading onto chromatin (HSA-2470946; *p* = 0.0381) (RAD21, SMC3), the positive regulation of macromolecule biosynthetic (GO: 001055; *p* = 0.0169) (FXR2, FOXA1, KDM6B, EP300, TP53, CEBPB, SP1, GATA2, SOX15, TCF4, FOXA2, FSHB, RXRA, HDAC2, MAFF, CTCF) and cellular biosynthetic processes (GO: 0031328; *p* = 0.0169) (FXR2, FOXA1, KDM6B, EP300, TP53, CEBPB, SP1, TCF4, GATA2, SOX15, FOXA2, FSHB, RXRA, HDAC2, MAFF, CTCF), cellular responses to stress (HSA-2262752; *p* = 0.0381) (KDM6B, EP300, TP53, CEBPB, SP1, MAFK, CHD9, RXRA), etc.

**Figure 4:**
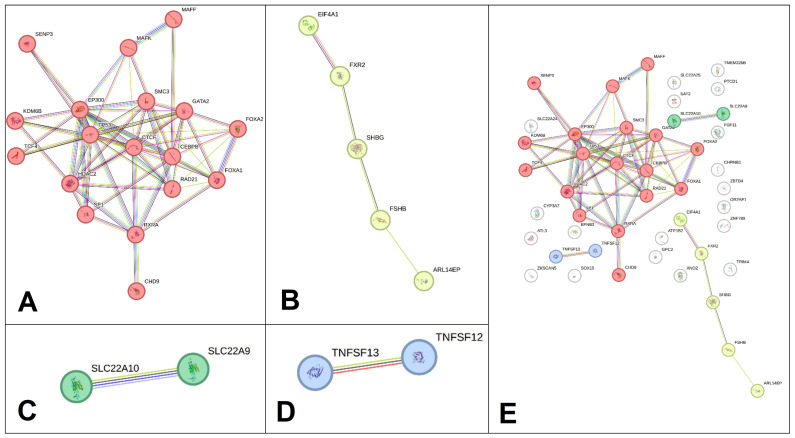
Clusters of a network of protein interactions linked with development of EH (STRING data): (**A**) cluster 1 (indicated in red); (**B**) cluster 2 (indicated in yellow); (**C**) cluster 3 (indicated in green); (**D**) cluster 4 (indicated in blue); (**E**) four clusters in total.

Based on the clustering procedure performed in the STRING program (k-means clustering technology was applied), four clusters were identified (Figure 4), which included 27 proteins under consideration; 19 proteins in the figure occupy “independent” positions and are not included in any of the four clusters formed. The first cluster, the most numerous (Figure 4A), includes 18 proteins such as CEBPB, CHD9, CTCF, EP300, FOXA1, FOXA2, GATA2, HDAC2, KDM6B, MAFF, MAFK, RAD21, RXRA, SMC3, SENP3, SP1, TCF4, and TP53. EP300, CEBPB and CTCF were involved in the largest number of interactions (12, 11 and 11 interactions, respectively), and the most pronounced paired protein interactions (score ≥ 0.99) were demonstrated by regulatory proteins TP53-EP300, SP1-EP300, SMC3-RAD21, RAD21-CTCF, EP300-CEBPB, TP53-SP1, and TP53-HDAC2. Interactions of 1-st cluster proteins are important in the following processes:

(a) The regulation of gene transcription, including transcription regulation by RNA polymerase II (GO: 0006357; *p* = 5.31 × 10^−7^) (FOXA1, KDM6B, EP300, TP53, RAD21, CEBPB, SP1, MAFK, GATA2, TCF4, FOXA2, RXRA, HDAC2, MAFF, CTCF), chromatin organization (GO: 0006325; *p* = 6.85 × 10^−5^) (FOXA1, KDM6B, EP300, TP53, CHD9, FOXA2, HDAC2, CTCF) and remodeling (GO: 0006338; *p* = 0.0291) (FOXA1, KDM6B, CHD9, HDAC2), the regulation of peptidyl-lysine acetylation (GO: 2000756; *p* = 0.0147) (GATA2, HDAC2, CTCF), histone acetyltransferase binding (GO: 0035035; *p* = 0.0004) (TP53, CEBPB, SP1), histone deacetylase binding (GO: 0042826; *p* = 0.0009) (TP53, CEBPB, SP1, HDAC2), etc.
(b) The processes of embryogenesis and development: Embryo development (GO: 0009790; *p* = 6.85 × 10^−5^) (FOXA1, KDM6B, EP300, TP53, CEBPB, GATA2, FOXA2, HDAC2, MAFF), the regulation of the developmental process (GO: 0050793; *p* = 0.0058) (FOXA1, TP53, CEBPB, SP1, GATA2, TCF4, FOXA2, RXRA, HDAC2, MAFF), epithelium development (GO: 0060429; *p* = 0.0077) (FOXA1, KDM6B, EP300, TP53, CEBPB, GATA2, HDAC2), epithelial cell differentiation (GO: 0030855; *p* = 0.0341) (FOXA1, KDM6B, CEBPB, GATA2, HDAC2) and epithelial cell maturation (GO: 0002070; *p* = 0.0405) (FOXA1, GATA2), the positive regulation of cell–cell adhesion mediated by cadherin (GO: 2000049; *p* = 0.0152) (FOXA1, FOXA2), the regulation of epithelial to mesenchymal transition (GO: 0010717; *p* = 0.0203) (FOXA1, FOXA2, HDAC2), regulation of the transforming growth factor β (TGFβ) receptor signaling pathway (GO: 0017015; *p* = 0.0405) (EP300, TP53, HDAC2), fat cell differentiation (GO: 0045444; *p* = 0.0321) (EP300, CEBPB, GATA2), etc.
(c) Metabolic processes: The regulation of macromolecule metabolic process (GO: 0060255; *p* = 0.0010) (FOXA1, KDM6B, EP300, TP53, RAD21, CEBPB, MAFK, GATA2, SMC3, TCF4, SP1, FOXA2, RXRA, HDAC2, MAFF, CTCF), the negative regulation of cellular metabolic process (GO: 0031324; *p* = 0,0003) (FOXA1, EP300, TP53, CEBPB, MAFK, GATA2, FOXA2, RXRA, HDAC2, MAFF, CTCF), the regulation of glucose metabolic process (GO: 0010906; *p* = 0.0253) (EP300, TP53, FOXA2), cellular responses to stress (GO: 0033554; *p* = 0.0476) (KDM6B, EP300, TP53, RAD21, CEBPB, SMC3, HDAC2), etc.

The 2-nd cluster (Figure 4B), consisting of five proteins (ARL14EP, EIF4A1, FSHB, FXR2, SHBG), is characterized by the most pronounced EIF4A1-FXR2 interactions (score = 0.705) and is involved in the processes of FSH [follicle-stimulating hormone complex (GOCC: 0016914; *p* = 0.0043)]. The 3-rd cluster is represented by only one pair interaction, SLC22A10-SLC22A9 (score = 0.448) (Figure 4C), the biological pathways of which are not known to date. The 4-th cluster also includes one pair interaction, TNFSF12-TNFSF13 (score = 0.938) (Figure 4D), which, according to the materials of the local STRING network, is linked with TNF receptor superfamily members mediating the non-canonical NF-kB pathway and transient hypogammaglobulinemia (CL: 15905; *p* = 0.0025).

So, our detailed analysis of the EH-correlated protein interactions (with the allocation of four clusters) allowed us to establish EH-significant biological pathways that involve the SNPs–genes–proteins we are considering, such as the development, differentiation and maturation of the epithelium, the TGFβ pathway, fat cell differentiation, gene expression, metabolic process regulation, etc.

## 4. Discussion

In this report, for the first time, it was found that the SNPs that determine the level of sex hormones are EH-associated: minor polymorphic variants rs11031002 (for the A—OR allele = 0.45–0.50) and rs11031005 (for the C—OR allele = 0.05–0.53) of the *FSHB* gene were associated with a low risk of developing the disease (1.31% and 1.32% of the disorder variance are determined accordingly), and the TT*rs11031002-rs11031005 *FSHB* haplotype, at a level of statistical significance exceeding the GWAS “standard” (*p* = 1 × 10^−11^), increases EH risk by more than 2.5 times (OR = 2.84).

Previously conducted GWASs showed the important role of SNP T>A rs11031002 and T>C rs11031005 of the *FSHB* gene in the formation of an organism’s “hormonal profile”: rs11031002 was associated with the level of LH [18] and CGA, while FSHB [21] and rs11031005 were associated with the concentration of FSH [18], total and bioavailable testosterone [19,22,24], and the testosterone/SHBG ratio (FAI) [24]. These SNPs were also associated (GWAS data) with the formation of such hormone-significant phenotypes/diseases as polycystic ovary syndrome (rs11031002 [54] and rs11031005 [55]), bone mineral density (rs11031002) [56], endometriosis in combination with migraine (rs11031005) [57], age of menarche (rs11031005) [58] and menopause (rs11031005) [59], and ovarian cysts (rs11031005) [60].

In the work of Garitazelaia et al., who performed a Mendelian randomization (MR) of GWAS data, it was shown that the loci T>A rs11031002 and T>C rs11031005 *FSHB* were pleiotropically associated with both endometriosis and with such signs characterizing the female reproductive system as the level of sex hormones (β = −1.03 and β = 0.95, respectively) and the age of menopause (β = −4.04 for rs11031005) [61]. An earlier genetic study on endometriosis (the sample included 1376 women, 395 of whom had endometriosis, with 981 controls) in the population examined in this study (Central Chernozem region of Russia) showed the protective value of allelic variants A rs11031002 (OR = 0.60–0.68) and C rs11031005 (OR = 0.65–0.66), as well as the risk role of the TT*rs11031002-rs11031005 haplotype (OR = 2.03) in the formation of the disease [43], which is completely consistent with our data on the protective role of the minor SNP alleles T>A rs11031002 and T>C rs11031005 *FSHB* in EH formation. It should be noted that the SNPs T>A rs11031002 and T>C rs11031005 *FSHB* are located at a distance of 95 nucleotide pairs and are strongly interconnected (in the sample we studied, the r^2^ index for these two loci is 0.62, and, according to Haploreg data, r^2^ = 0.79/D’ = 0.99), and therefore their genetic effects can largely “overlap” and be “shared”.

Numerous literature data presented both in experimental works (including those based on GWASs) [59,61,62,63,64,65,66,67,68,69,70,71] and in review articles [72,73,74] convincingly demonstrate a pronounced association with various hormone-dependent signs (diseases) in a sufficiently large number of other SNPs (rs11031006, rs10835638, rs74485684, rs1782507, rs11031010, rs555621, etc.) located in the *FSHB* promoter region (0.21–40 Kb of the 5′ region of the *FSHB* gene).

In more than ten different GWASs, the association of rs11031006 *FSHB* with reproductively significant phenotypes such as FSH [70], LH and polycystic ovary syndrome [64], FSH and the birth of dizygotic twins [65], age at menarche [59,66] and menopause [62], characteristics of the menstrual cycle (duration, presence of excessive, frequent and irregular menstruation) and ovariectomy (bilateral) [67], uterine fibroids [71], uterine fibroids and copious menstrual bleeding [68], endometriosis, age at menarche and duration of the menstrual cycle (pleiotropic connections revealed by the MR method) [61], and polycystic ovary syndrome [63,69] has been shown. It should be noted that rs11031006 is located at a distance of 72 pairs of nucleotides from the rs11031005 we are studying, and is strongly linked to it at r^2^ = 1/D’ = 1, and at a distance of 267 pairs of nucleotides from the rs11031002 we are considering, and is also strongly linked to it at r^2^ = 0.79/D’ = 0.99.

An equally important biomedical significance (link with hormone-dependent reproductively significant indicators) has been shown in a number of studies for the polymorphism rs10835638 *FSHB* (location—210 bp 5′ of this gene), located at a distance of 26 kb and 37.1 kb from the rs11031005 and rs11031002 studied by us, respectively, and strongly linked to them when r^2^ = 0.62/D’ = 0.79 and r^2^ = 0.74/D’ = 0.95, respectively. Thus, in the work of Ruth et al., rs10835638 was found to be associated with LH, menstrual cycle duration, menopause age, and infertility development in women with endometriosis [18]. In a study by Bianco et al., the association of this SNP with LH levels was found in patients with endometriosis suffering from infertility [75]. According to Rull et al., polymorphism rs10835638 (−211 G>T *FSHB*) was associated with the concentration of FSH and LH in women with amenorrhea and infertility [76]. This genetic variant showed significant associations with LH levels, response to controlled ovarian hyperstimulation, the number of antral follicles, eggs, and embryos obtained [77], polycystic ovary syndrome [69], and the level of FSH in idiopathic male infertility [78].

The relationship of other polymorphisms localized in the regulatory regions of the *FSHB* gene with hormone-dependent phenotypes is also indicated in a significant number of different scientific publications: age at menarche (rs1782507 [8.7 kb 5′ of the *FSHB*], rs555621 [16 kb 5′ of the *FSHB*]) [79], menarcheal age, LH levels and polycystic ovary syndrome (rs11031010 [12 kb 5′ of the *FSHB*]) [79,80], menopausal age, testosterone concentration, LH level and LH/FSH ratio (rs12294104 [23 kb 3′ of the *C11orf46*], located at a distance of 156.5 kb and 167.6 kb from the rs11031005 and rs11031002 studied by us, respectively, and strongly linked to them at r^2^ = 0.37/D’ = 0.66 and r^2^ = 0.45/D’ = 0.80, respectively) [19,81,82,83,84], uterine fibroids (rs76959488 [17 kb 3′ of the *C11orf46*], located at a distance of 149.9 kb and 161.1 kb from the rs11031005 and rs11031002 studied by us, respectively, and strongly linked to them at r^2^ = 0.42/D’ = 0.68 and r^2^ = 0.51/D’ = 0.83, respectively) [60], and endometriosis (rs74485684 [10 kb 5′ of the *FSHB*]) [85]. It is important to note that, for a sample of women from the population of the Central Chernozem region of Russia (studied in this work), the associations of *FSHB* promoter region polymorphisms (8.7–16 kb 5′ of this gene)—rs1782507, rs11031010, and rs555621—with hormone-dependent signs/diseases such as newborn weight (rs1782507, rs555621) [86], BMI of adult women (rs555621) [87], uterine fibroids [88], endometriosis [89], and endometrial hyperplasia [15] were previously shown.

Thus, based on our results and the above-mentioned numerous literature data, it can be argued that functionally significant polymorphic loci of the *FSHB* gene promoter region (rs11031002, rs11031005, rs11031006, rs10835638, rs74485684, rs1782507, rs12294104, rs11031010, rs76959488, rs555621) play a key role in determining the hormonal status of a female organism and the formation of hormone-dependent phenotypes (normal signs and diseases), which allows us to consider this gene as a “syntropic gene” for a variety of hormone-related signs/pathologies. This opens up broad prospects for both further medical and genetic studies of the SNPs of the *FSHB* gene in relation to other hormone-dependent phenotypes (insufficiently studied to date) and the use of these gene polymorphisms in practical medicine (predictive testing) as genetic markers of an increased risk of developing hormone-dependent diseases.

The biomedical basis for the involvement of polymorphic loci T>A rs11031002 and T>C rs11031005 *FSHB* in EH formation may be due to the following putative mechanisms. First, minor allelic variants of the SNPs T>A rs11031002 and T>C rs11031005, which are of protective importance in EH development (our data), were associated with high levels of LH [18] and low concentrations of CGA;FSHB [21] and FSH [18], which may be essential in EH development.

The literature data clearly indicate the primary role of hormonal factors in EH development [5,6,7]. It is indicated that an increase in FSH and the FSH/LH ratio, a decrease in LH, an imbalance in the estrogen–progesterone system (absolute hyperestrogenism; normal estrogen content with a lack of progesterone), etc., predispose to EH development [5,6,7]. An imbalance in the levels of FSH, LH and the FSH/LH ratio may cause the appearance of anovulatory cycles in a woman [90,91]. Chronic anovulation (especially recorded during perimenopause) is considered an important hormone-related risk factor for EH development [5,6]. In anovulatory cycles, the level of estrogens that stimulate endometrial proliferation is dominant without the counteracting effects of the progesterone (anti-proliferative effect on endometrial cells) produced by the corpus luteum after ovulation. This imbalance in the estrogen–progesterone system leads to continued proliferation of the endometrium, which leads to higher risks of developing EH [6].

In the work of Hambridge et al. (250 healthy premenopausal women were studied), it was shown that, in women with one anovulatory cycle, the peak concentration of LH and the levels of sex hormones (progesterone, estradiol) were lower (by 38%, 22% and 25%, respectively) compared with women with two ovulatory cycles [91]. The authors found the most pronounced deviations in the level of progesterone (reduced by more than 4 times) and estradiol (−60%) in women with two anovulatory cycles when compared with women with two ovulatory cycles [91]. In the Burger et al. study, it has been demonstrated that anovulatory cycles in women over 45 years of age are usually characterized by an increased level of FSH with a low inhibin content [90]. This is based, according to the authors, on an age-related decrease in the number of primordial ovarian follicles (up to 100), which is reflected in a decrease in the number of small antral follicles (the site of inhibin B production), which causes a decrease in the formation of inhibin B (it is a repressor of FSH synthesis), and this, in turn, leads to an increase in the FSH level (ensures maintenance of circulating estradiol levels) [90]. At the same time, an increased level of FSH and a significantly reduced progesterone concentration can lead to abnormal endometrial growth [18] and can thus be risk factors for EH formation [5].

Importantly, polymorphisms strongly linked to the EH-associated loci under consideration (T>A rs11031002 and T>C rs11031005 *FSHB*) also have significant correlations with the levels of LH and FSH (rs11031006 [64,65,70], rs10835638 [18,75,76,77,78], rs11031010 [80], rs12294104 [84]) and, as a result, may cause LH/FSH-mediated phenotypic effects on SNP T>A rs11031002 and T>C rs11031005 *FSHB* genes in EH.

It should be noted that there is also data on the relationship of the FSH-reducing *FSHB* polymorphism (rs10835638) with late menarche (and, accordingly, on the relationship of the FSH-increasing genetic variant with early menarche) [18], which fully corresponds to both modern literary ideas about the role of FSH and the age at menarche in EH pathophysiology (high FSH levels and early menarche are risk factors for disease development [5]) and our data on the association of FSH-reducing minor allelic variants of SNPs T>A rs11031002 and T>C rs11031005 *FSHB* (rs11031005 *FSHB* and strongly associated loci [rs1782507, rs11031006, rs555621, rs11031010] are also associated with the age at menarche [58,59,61,66,79,80]) with a low EH risk (OR < 1).

Secondly, one of the potential mechanisms determining the relationship of the T>C rs11031005 locus of the *FSHB* with EH may be its effect on the content of total and bioavailable testosterone in the organism [19,22,24], as well as on the testosterone/SHBG (FAI) ratio [24]: a minor allele C rs11031005 (a protective factor for EH development according to our data) is associated with higher concentrations of total and bioavailable testosterone [19,22,24] and a low FAI [24].

Modern literature data based on a large number of experimental studies indicate a significant effect of androgens (testosterone, dehydroepiandrosterone (DHEA), androstenedione, dihydrotestosterone (DHT)) on the physiology of the female reproductive system [8,9]. These effects of androgens can be independent (due to binding to their specific receptors (AR) and can influence the expression of target genes (for example, targeted genes in the endometrium are *CITED2*, *ACSS2*, *PPFIBP2*, *MAOA*, etc. [92]) indirectly (due to the effect on estrogens and progesterone), which leads to a complex network of interactions of steroid hormones and plays an important regulatory role in the menstrual cycle, endometrial biology, and follicle development in the ovaries [9]. In women, 80% of androgens bind to SHBG, 19% bind to serum albumin, and only 1% are free; free androgens are the only active androgens [8]. In addition, androgens are converted by aromatase into estrogens, and, due to them, they can already realize their biological effects [8].

Testosterone, by increasing the expression of insulin-like growth factor-1, stimulates the growth and maturation of primordial follicles, oocyte metabolism, follicle recruitment and oocyte extraction, and enhances follicle response to FSH [9,93,94]. At the same time, in the late stages of follicle development, androgens inhibit follicle growth and estrogen production and stimulate the apoptosis of granulosa cells and the transition to follicle maturation [95], as well as the development of the antrum cavity [96]. In addition, androgen signaling in the ovaries stimulates the formation of yellow bodies by enhancing the expression of the FSH receptor, thus also having an indirect stimulating effect on progesterone production [97,98]. In experimental models of transgenic animals, it has been convincingly shown that, when androgen receptors are knocked out, significant disorders in the ovaries are observed (impaired follicle development, longer estrous cycles, fewer yellow bodies, increased follicle atresia, impaired egg extraction, etc.) [9,99], which is of paramount importance in EH pathogenesis [5].

The data presented in the literature on the effect of testosterone on endometrial cell proliferation are very interesting. AR expression is believed to occur predominantly in endometrial stromal cells, and it increases at the end of the proliferative–early secretory phases [9,100]. On the one hand, in vivo experiments (mice with ovariectomy and transgenic mice) have shown direct links between the administration of testosterone, DHT, and endometrial cell proliferation (due to the activation of signaling pathways of insulin-like growth factor-1) [9,101,102]. On the other hand, a number of in vitro studies have demonstrated the inhibitory effects of androstenedione, testosterone, and DHT on the proliferation of human endometrial cells (both stromal and glandular) [92,103,104,105] in contrast to the proliferative effects of estrogens [9]. It has also been shown that the use of exogenous testosterone (above normal physiological levels) for the treatment of women led to endometrial atrophy and decreased cell proliferation [106,107]. In vitro experiments have shown that DHT significantly reduces the activity of caspases in human endometrial stromal cells, which is important for stromal–glandular epithelial interactions and the regulation of androgen-dependent cellular apoptosis [92].

Thus, the above literature materials contain sufficiently convincing arguments to substantiate the protective effects of minor allelic variants of SNPs T>A rs11031002 and T>C rs11031005 *FSHB* (found in our study), related according to the GWAS data [19,22,24] with increased levels of total and bioavailable testosterone in the organism (including women). It should be noted that polymorphisms strongly linked to the EH-related loci under consideration (T>A rs11031002 and T>C rs11031005) demonstrate significant associations with both testosterone levels (rs12294104 [19]) and with testosterone-significant diseases such as polycystic ovary syndrome (rs11031006 [63,64,69], rs10835638 [69], rs11031010 [80]).

Thirdly, the pathogenic effects of SNPs T>A rs11031002 and T>C rs11031005 *FSHB* in relation to EH can be realized through significant risk factors for the development of this disease, with which these loci and strongly linked polymorphisms are associated. According to the literature, important risk factors for EH development are earlier menarche and late menopause, which cause an increase in the duration of the effect of estrogens on the endometrium during a woman’s life [5,7]. A number of GWASs show associations of the EH-causal SNP T>C rs11031005 *FSHB* with the age at menarche [58] and menopause [59,61]. There are also numerous materials (more than 10 studies, including GWASs) on the association of loci strongly linked to T>A rs11031002 and T>C rs11031005 *FSHB* with such risk factors for EH as menarcheal age (rs11031006 [59,61,66], rs1782507 [79], rs555621 [79], rs11031010 [79,80]), the age at menopause (rs11031006 [62], rs10835638 [18], rs12294104 [81,82,83]), and the BMI of adult women (rs555621) [87].

Fourthly, the involvement of SNPs T>A rs11031002 and T>C rs11031005 *FSHB* in EH formation may be related to the phenotypic effects of the genes whose functionality (expression, etc.) they control. The data obtained by us in silico indicate significant regulatory (epigenetic) effects (due to the coordination of DNA interaction with six transcription factors—Zfp281, Otx2, Pou6f1, HDAC2, Zfp105, and Pou2f2) of these polymorphisms on the promoter region of the *FSHB* gene (26–37 kb 5′ region of this gene) and the association with the expression of *ARL14EP* in more than 10 different organs, including those that are significant for EH pathophysiology—subcutaneous fat, the thyroid gland, etc. (EH-protective alleles of these SNPs [A rs11031002, C rs11031005] were associated with higher transcriptional activity of this gene). It should be noted that, according to the literature, the *ARL14EP* gene can be expressed in various organs of the female reproductive system (ovaries, uterus) [74], and the resulting protein ARL14EP (ADP ribosylation factor-like GTPase 14 effector protein) participates in various interactions with ACTß (β-actin), ARL14 (ADP-ribosylation factor-like 14), and MYO1E (actin-based motor protein myosin 1E) and controls the export of major molecules of histocompatibility class II by binding to the actin network [74].

The *FSHB* gene controls the formation of the β-subunit of FSH, whose interaction with the α-subunit (common to all pituitary and placental glycoprotein hormones) forms the FSH dimer; only FSH-β gives FSH-specific biological activity, and therefore the synthesis stage of this chain is the stage regulating the rate of “appearance” of biologically active FSH in the organism [108]. FSH is of fundamental importance to the normal functioning of the hypothalamic–pituitary–gonadal system of an organism and, interacting with its specific receptors (FSHRs), plays a key role in reproductively significant processes such as follicle development, egg maturation, the regulation of steroid hormone formation, granulosa cell growth, and the induction of androgen-converting enzyme (aromatase) synthesis [75]. The formation of FSH in adenohypophysis is under the direct control (positive relationship) of the gonadotropin-releasing hormone of the hypothalamus and is regulated by sex hormones (estrogens, progesterone, testosterone), glucocorticoids and other factors (activin, follistatin, etc.) [108]. It is noted that an increased level of FSH can lead to abnormal endometrial growth [18], which, in turn, leads to an increased EH risk [5].

It should be noted that the results obtained in this work on the significant involvement of two SNP loci, T>A rs11031002 and T>C rs11031005, of the *FSHB* gene in EH pathogenesis (the presence of pronounced main effects and associations in haplotypes at the GWAS level) not only have important fundamental significance (understanding the role of a specific genetic determinant in the formation of the disease), but may also have important practical significance in the future. These polymorphisms of the *FSHB* gene, after conducting replicative studies in other ethno-territorial population groups, as well as after conducting additional clinical and associative studies among EH patients with atypia (endometrial intraepithelial neoplasia [EIN]), can be used as potential biomarkers in order to predict the risk of EIN in patients with EH without atypia. Currently, there is an obvious “request” from practical medicine (gynecology/oncology) to develop immunohistochemical/molecular/genetic biomarkers that could reliably/reproducibly distinguish between normal/benign/precancerous/malignant endometria and indicate/predict the transition between these four groups [5]. To date, no biomarker has been found that fully meets these “requirements”, and an active search continues [5,109,110,111,112]. Among the markers that could be used to solve the above tasks are biomarkers such as PAX2 (paired box gene 2), PTEN (phosphatase and tensin homolog), tumor protein p53, HAND2 (heart and neural crest derivatives expressed transcript 2), MMR (DNA mismatch re-pair), β-catenin, ERa and ERb (estrogen receptors alpha and beta), PRs (progesterone receptors), COX-2 (Cyclooxygenase-2) and a number of others [5,109,110,113]. One promising biomarker may be PAX2, which is a member of a large family of paired box genes and participates in the regulation of gene expression in embryogenesis, while also acting as a protooncogene by regulating cell proliferation/survival/apoptosis [5]. It is assumed that the loss of PAX2 expression occurs at an early stage of the endometrial carcinogenesis process and leads to the development of EIN [114]. According to the 2020 WHO recommendations/classification, in addition to the main morphological parameters, an insufficient expression of PAX2, PTEN, and MMR is a desirable criterion for the diagnosis of EIN [1]. The results of a number of studies show that the use of PAX2 in various combinations with other markers (PAX2, PTEN, β-catenin [115,116], PAX2, HAND2, PTEN [110]) is an effective additional tool in the diagnosis of EIN [113]. In this regard, there is an obvious need for further active experimental research in this area to find effective immunohistochemical/molecular/genetic biomarkers that are included in routine clinical practice [113].

A number of limitations of the present study should be noted: (a) women of the control group who did not have pelvic organ disease symptoms, according to anamnestic and clinical/ultrasound examination, had no morphological evidence of the absence of EH, which makes some misclassification of the control group possible; (b) the results obtained in the work need to be confirmed in an independent cohort and are therefore preliminary.

## 5. Conclusions

Genetic determinants of sex hormone levels, involved in numerous hormone-mediated molecular pathways (regulation of gene transcription, processes of embryogenesis and development, regulation of metabolism, etc.), are associated with EH.

## Figures and Tables

**Table 1:** Characteristics of participants from the case and control groups.

Parameters	Cases	Controls	*p*
	(n = 520)	(n = 973)	
	$\bar{X}$ ± SD/% (n)	$\bar{X}$ ± SD/% (n)	
Age, years	41.78 ± 10.04	40.26 ± 8.53	>0.05
Height, m	1.66 ± 0.06	1.66 ± 0.06	>0.05
Weight, kg	73.67 ± 14.66	70.54 ± 13.25	**<0.001**
BMI, kg/m^2^	26.94 ± 5.56	25.22 ± 4.52	**<0.001**
Proportion of the participants by relative BMI, % (n):			
Underweight (<18.50)	2.69 (14)	3.60 (35)	**<0.001**
Normal weight (18.50–24.99)	35.00 (182)	54.98 (535)	
Overweight (25.00–29.99)	33.27 (173)	27.85 (271)	
Obese (>30.00)	29.04 (151)	13.57 (132)	
Family history of benign proliferative diseases of the uterus *	32.88 (171)	17.06 (166)	**<0.001**
Married	85.76 (446)	85.92 (836)	>0.05
Smoking (yes)	15.96 (83)	17.06 (166)	>0.05
Drinking alcohol (≥7 drinks per week)	3.27 (17)	3.08 (30)	>0.05
Oral contraceptive use	9.88 (51)	10.07 (98)	>0.05
Age at first oral contraceptive use (mean, years)	23.26 ± 2.32	23.61 ± 2.34	>0.05
Age at menarche and menstrual cycle			
Age at menarche, years	13.34 ± 1.28	13.29 ± 1.26	>0.05
Proportion of the participants by relative age at menarche, % (n)			
Early (<12 years)	5.23 (27)	6.17 (60)	>0.05
Average (12–14 years)	83.53 (431)	80.06 (779)	
Late (>14 years)	11.24 (58)	13.77 (134)	
Duration of bleeding, menstrual (mean, days)	5.13 ± 1.39	4.96 ± 0.95	>0.05
Menstrual cycle length (mean, days)	27.94 ± 2.15	28.18 ± 2.25	>0.05
Reproductive characteristic			
Age at first birth (mean, years)	21.12 ± 2.37	21.69 ± 3.48	>0.05
No of gravidity (mean)	2.84 ± 2.45	2.42 ± 1.53	>0.05
No of births (mean)	1.23 ± 0.88	1.50 ± 0.66	**<0.001**
No of spontaneous abortions (mean)	0.22 ± 0.53	0.23 ± 0.50	>0.05
No of induced abortions (mean)	1.35 ± 1.55	0.66 ± 0.97	**<0.001**
No of induced abortions			
0	37.88 (197)	58.99 (574)	**<0.001**
1	25.38 (132)	23.74 (231)	
2	18.85 (98)	10.18 (99)	
3	8.65 (45)	5.45 (53)	
≥4	9.23 (48)	1.64 (16)	
History of infertility	11.92 (62)	5.14 (50)	**<0.001**
Gynecological pathologies			
Cervical disorders	26.54 (138)	25.18 (245)	>0.05
History of sexually transmitted disease	26.35 (137)	26.93 (262)	>0.05
Chronic endometritis	14.04 (73)	5.65 (55)	**<0.001**
Chronic inflammation of adnexa	34.23 (178)	31.96 (311)	>0.05
Uterine leiomyoma	51.54 (268)	-	-
Endometriosis	35.19 (183)	-	-
Adenomyosis	20.58 (107)	-	-

Note: *—Mother had endometrial hyperplasia, uterine leiomyoma, endometriosis, or adenomyosis, *p* values < 0.05 are shown in bold.

**Table 2:** Associations of the studied gene polymorphisms with endometrial hyperplasia.

Chr	SNP	Minor Allele	Gene	n	Allelic Model				Additive Model				Dominant Model				Recessive Model			
					OR	95%CI		*p*	OR	95%CI		*p*	OR	95%CI		*p*	OR	95%CI		*p*
						L95	U95			L95	U95			L95	U95			L95	U95	
7	rs148982377	C	*ZNF789*	1451	1.21	0.86	1.70	0.266	1.25	0.87	1.79	0.232	1.22	0.83	1.78	0.317	4.21	0.43	41.58	0.219
7	rs34670419	T	*ZKSCAN5*	1450	0.96	0.64	1.43	0.839	1.02	0.67	1.55	0.942	1.03	0.66	1.60	0.911	0.80	0.07	8.93	0.854
11	rs11031002	A	*FSHB*	1427	**0.50**	**0.38**	**0.66**	**5 × 10^−7^**	**0.45**	**0.33**	**0.61**	**4 × 10^−7^**	**0.43**	**0.31**	**0.59**	**3 × 10^−7^**	0.33	0.07	1.45	0.141
11	rs11031005	C	*FSHB*	1452	**0.52**	**0.40**	**0.68**	**1 × 10^−6^**	**0.51**	**0.38**	**0.69**	**8 × 10^−6^**	**0.53**	**0.39**	**0.73**	**7 × 10^−5^**	**0.05**	**0.01**	**0.39**	**0.005**
11	rs112295236	G	*SLC22A10*	1440	1.22	0.88	1.70	0.235	1.28	0.89	1.85	0.186	1.31	0.90	1.90	0.156	0.01	0.00	inf	0.999
12	rs117585797	A	*ANO2*	1428	0.78	0.46	1.32	0.357	0.90	0.51	1.58	0.707	0.91	0.51	1.61	0.749	0.01	0.00	inf	0.999
16	rs117145500	C	*CHD9*	1427	1.05	0.81	1.36	0.695	0.94	0.71	1.24	0.650	0.88	0.65	1.19	0.404	1.98	0.67	5.90	0.218
17	rs727428	T	*SHBG*	1440	0.96	0.82	1.13	0.638	0.94	0.78	1.12	0.460	0.94	0.74	1.20	0.640	0.87	0.61	1.23	0.429
17	rs1641549	T	*TP53*	1430	0.91	0.76	1.09	0.311	0.92	0.76	1.12	0.418	0.89	0.70	1.14	0.349	0.96	0.60	1.54	0.865

Note: OR—odds ratio; 95% CI—95% confidence interval; all results were obtained after adjustment for covariates; *p*_perm_ values < 0.0125 are shown in bold.

**Table 3:** Haplotypes of polymorphic loci of the *FSHB* gene and EH risk.

SNP		Frequency		OR	*p*	*p*_adj-perm_
rs11031002	rs11031005	EH (n = 520)	Controls (n = 973)			
A	C	**0.067**	**0.113**	**0.68**	**0.013**	**0.036**
T	C	**0.009**	**0.023**	**0.18**	**2 × 10^−5^**	**6 × 10^−4^**
A	T	**0.005**	**0.022**	**0.03**	**1 × 10^−10^**	**1 × 10^−6^**
T	T	**0.919**	**0.842**	**2.84**	**1 × 10^−11^**	**1 × 10^−6^**

Note: OR—odds ratio; *p*—significance level; the results were obtained through the logistic regression analysis with adjustment for covariates; statistically significant results are highlighted in bold, taking into account the permutation test (1000 permutations were performed).

**Table 4:** SNP × SNP interactions significantly associated with EH.

N	SNP × SNP Interaction Models	NH	*beta*H	WH	NL	*beta*L	WL	*p*_adj-perm_
Two-order interaction models (*p* < 7.57 × 10^−7^)								
1	rs11031002 *FSHB* × rs11031005 *FSHB*	1	1.03	43.71	3	−0.91	32.86	<0.001
2	rs11031002 *FSHB* × rs112295236 *SLC22A10*	2	0.85	27.28	1	−0.83	27.28	<0.001
3	rs117145500 *CHD9* × rs11031002 *FSHB*	1	0.57	19.54	2	−0.85	25.32	<0.001
4	rs11031002 *FSHB* × rs148982377 *ZNF789*	2	0.80	24.71	2	−0.81	24.71	<0.001
5	rs11031002 *FSHB* × rs117585797 *ANO2*	1	0.74	24.46	1	−0.83	24.46	<0.001
Three-order interaction models (*p* < 2.52 × 10^−10^)								
1	rs11031002 *FSHB* × rs1641549 *TP53* × rs11031005 *FSHB*	1	0.52	19.17	6	−1.29	43.86	<0.001
2	rs11031002 *FSHB* × rs117585797 *ANO2* × rs11031005 *FSHB*	1	0.92	40.02	3	−0.94	33.68	<0.001
3	rs11031002 *FSHB* × rs112295236 *SLC22A10* × rs11031005 *FSHB*	2	1.04	44.24	3	−0.94	32.90	<0.001
4	rs11031002 *FSHB* × rs727428 *SHBG* × rs11031005 *FSHB*	2	0.74	31.17	3	−2.67	42.83	<0.001
5	rs11031002 *FSHB* × rs11031005 *FSHB* × rs148982377 *ZNF789*	2	0.99	41.11	3	−0.95	26.43	<0.001
Four-order interaction models (*p* < 2.05 × 10^−10^)								
1	rs11031002 *FSHB* × rs1641549 *TP53* × rs11031005 *FSHB* × rs34670419 *ZKSCAN5*	1	0.52	19.73	5	−1.30	40.42	<0.001
2	rs11031002 *FSHB* × rs117585797 *ANO2* × rs112295236 *SLC22A10* × rs11031005 *FSHB*	2	0.93	40.53	3	−0.94	32.20	<0.001
3	rs11031002 *FSHB* × rs117585797 *ANO2* × rs727428 *SHBG* × rs11031005 *FSHB*	2	0.73	32.46	3	−3.03	44.06	<0.001
4	rs11031002 *FSHB* × rs117585797 *ANO2* × rs11031005 *FSHB* × rs148982377 *ZNF789*	2	0.89	37.68	2	−2.53	45.99	<0.001
5	rs11031002 *FSHB* × rs112295236 *SLC22A10* × rs727428 *SHBG* × rs11031005 *FSHB*	3	0.74	33.62	3	−2.99	42.60	<0.001
6	rs11031002 *FSHB* × rs112295236 *SLC22A10* × rs11031005 *FSHB* × rs148982377 *ZNF789*	3	0.96	40.89	2	−2.49	44.16	<0.001

Note: NH—number of significant high-risk genotypes in the interaction; *beta*H—regression coefficient for high-risk exposition in the step2 analysis; WH—Wald statistic for high-risk category; NL—number of significant low-risk genotypes in the interaction; *beta*L—regression coefficient for low-risk exposition in the step2 analysis; WL—Wald statistic for low-risk category; *p*_adj-perm_—permutation *p*-value for the interaction model (1000 permutations). The results were obtained using the MB-MDR method with adjustment for covariates. The different levels of 61 genotype combinations [13 (21.31%)—two loci, 21 (34.43%)—three loci, 27 (44.26%)—four loci] determining the EH risk were exploratorily modeled (Table S5). Moreover, among them, more than half of the genotype combinations (n = 38, 62.29%) were risky, and a smaller part (n = 23, 37.71%) was protective in the formation of the disease. The most pronounced phenotypic effects (different by the highest values of the *beta* index) were manifested by the following combinations of genotypes: rs11031002 × TA × rs1641549 × CT × rs11031005 × TT (*beta* = −3.82, *p* = 0.003), rs11031002 × TA × rs1641549 × CT × rs11031005 × TT × rs34670419 × GG (*beta* = −3.82, *p* = 0.003), rs11031002 × TA × rs112295236 × CC × rs11031005 × TT (*beta* = −3.56, *p* = 9 × 10^−9^), rs11031002 × TA × rs117585797 × CC × rs112295236 × CC × rs11031005 × TT (*beta* = −3.56, *p* = 9 × 10^−9^), rs11031002 × TA × rs727428 × CT × rs11031005 × TT (*beta* = −3.55, *p* = 0.000003), rs11031002 × TT × rs117585797 × CC × rs727428 × CC × rs11031005 × TT (*beta* = −3.55, *p* = 0.000002), rs11031002 × TA × rs112295236 × CC × rs727428 × CT × rs11031005 × TT (*beta* = −3.52, *p* = 0.000003) (protective value) and rs11031002 × TT × rs11031005 × TT (*beta* = 1.03, *p* = 3 × 10^−8^), rs11031002 × TT × rs117585797 × CC × rs11031005 × TT (*beta* = 0.92, *p* = 2 × 10^−10^), rs11031002 × TT × rs112295236 × CG × rs727428 × CT × rs11031005 × TT (*beta* = 0.82, *p* = 0.003), and rs11031002 × TT × rs117585797 × CC (*beta* = 0.74, *p* = 7 × 10^−7^) (risk value) (Table S5).

## Data Availability

The data generated in the present study are available from the corresponding author upon reasonable request.

## Supplementary Materials

The following supporting information can be downloaded at: https://www.mdpi.com/article/10.3390/life16050782/s1, Table S1: The regulatory potential of the studied SNPs; Table S2: The GWAS data about associations of the studied candidate genes polymorphisms with the level of sex hormones; Table S3: The allele and genotype frequencies of the studied SNPs in the endometrial hyperplasia and control groups; Table S4: Genotype combinations associated with EH; Table S5: Regulatory effects of the EH-associated loci and SNPs in high LD (r^2^ ≥ 0.80); Table S6: The eQTL effects of the EH-associated SNPs in various tissues/organs; Table S7: eQTL values of SNPs in high LD (r^2^ ≥ 0.80) with the RH-associated polymorphisms; Table S8: The sQTL effects of the EH-associated SNPs in various tissues/organs; Table S9: sQTL values of SNPs in high LD (r^2^ ≥ 0.80) with the EH-associated polymorphisms.

## Abbreviations

The following abbreviations are used in this manuscript:

EH	Endometrial hyperplasia
SNP	Single-nucleotide polymorphism
GWAS	Genome-wide studies
SHBG	Sex hormone-binding globulin
LH	Luteinizing hormone
FSH	Follicle-stimulating hormone
LD	Linkage disequilibrium
