# Supplementary material for: Polymorphism of the FSHB Gene Is Associated with Endometrial Hyperplasia
Source: Life (Basel). 2026 May 7;16(5):782. doi: 10.3390/life16050782 (PMC13208232; doi:10.3390/life16050782)
Supplement: Supplementary file 1 [file life-16-00782-s001.zip › Supplementary Table S2.pdf]

Supplementary Table S2. The GWAS data about associations of the studied candidate genes polymorphisms with the level of sex hormones.

SNP	Position (chr: hg38)	Phenotype	Association (significance) (affected allele)	Reference
rs148982377	7: 99477415	DHEAS	β = -0.255 ($p = 1.82 \times 10^{-14}$) (C)	[18]
		Bioavailable Testosterone (premenopausal women)	β = -0.05 ($p = 4.29 \times 10^{-13}$) (C)	[20]
		Androsterone sulfate levels	β = -1.50 ($p = 1.00 \times 10^{-337}$) (C)	[25]
		Epiandrosterone sulfate levels	β = -1.32 ($p = 4.00 \times 10^{-270}$) (C)	[25]
		Androstenediol (3beta,17beta) disulfate (1) levels	β = -0.84 ($p = 5.00 \times 10^{-116}$) (C)	[25]
		Androstenediol (3alpha, 17alpha) monosulfate (3) levels	β = -0.31 ($p = 5.00 \times 10^{-16}$) (C)	[25]
rs34670419	7: 99533211	Progesterone	β = -0.346 ($p = 6.09 \times 10^{-14}$) (T)	[18]
		Cortisol/DHEAS ratio	β = 0.721 (p = 2.35 × 10-8) (T)	[17]
		DHEAS	β = -0.780 ($p = 2.07 \times 10^{-9}$) (T)	[17]
		4-androsten-3beta,17beta-diol disulfate (1) levels	β = -0.39 ($p = 4.00 \times 10^{-28}$) (T)	[23]
		4-androsten-3beta,17beta-diol monosulfate (1) levels	β = -0.47 ($p = 1.00 \times 10^{-41}$) (T)	[23]
		4-androsten-3beta,17beta-diol monosulfate (2) levels	β = -0.46 ($p = 2.00 \times 10^{-32}$) (T)	[23]
rs11031002	11: 30193714	LH	β = 0.221 ($p = 3.94 \times 10^{-9}$) (A)	[18]
		Serum levels of protein CGA;FSHB	β = -0.162 ($p = 5.00 \times 10^{-20}$) (A)	[21]
rs11031005	11: 30204809	FSH	β = -0.232 ($p = 1.74 \times 10^{-8}$) (C)	[18]
		Total testosterone	β = 0.033 ($p = 7.2 \times 10^{-17}$) (C)	[19]
		Bioavailable testosterone	β = 0.023 ($p = 1.5 \times 10^{-10}$) (C)	[19]
		Total testosterone	β = 0.027 ($p = 7.22\times 10^{-9}$) (C)	[22]
		Free testosterone levels (women)	β =-0.031 ($p = 2.00 \times 10^{-10}$) (T)	[24]
		Free androgen index (women)	β =-0.031 ($p = 4.00 \times 10^{-10}$) (T)	[24]
rs112295236	11: 63147874	Progesterone	β = 0.255 ($p = 7.68 \times 10^{-12}$) (G)	[18]
		Bioavailable testosterone (in men and women combined)	β = 0.031 ($p = 2.3 \times 10^{-14}$) (G)	[19]
rs117585797	12: 5902324	Oestradiol	β = 0.624 ($p = 1.63 \times 10^{-8}$) (A)	[18]
rs117145500	16: 52913718	FAI	β = -0.276 ($p = 1.50 \times 10^{-8}$) (C)	[18]
rs727428	17: 7634474	SHBG	β = -0.126 ($p = 2.09 \times 10^{-16}$) (T)	[16]
		Bioavailable testosterone	β = 0.095 ($p = 8.3 \times 10^{-309}$) (T)	[19]
		Oestradiol	β = -0.041 ($p = 2.10 \times 10^{-10}$) (T)	[22]
		Free Testosterone (women)	β = -0.001 ($p = 5.00 \times 10^{-21}$) (C)	[20]

		SHBG (premenopausal women)	β = 7.25 ($p = 5.43 \times 10^{-109}$) (C)	[20]
		SHBG	β = -0.22 ($p = 2.00 \times 10^{-16}$) (T)	[26]
		Bioavailable testosterone (postmenopausal women)	β = -0.019 ($p = 3.09 \times 10^{-25}$) (C)	[20]
		Bioavailable testosterone (women)	β = -0.021 ($p = 1.03 \times 10^{-48}$) (C)	[20]
rs1641549	17: 7671457	SHBG	β = -0.127 ($p = 1.21 \times 10^{-15}$) (T)	[18]

Note: Chr–chromosome; DHEAS–dehydroepiandrosterone sulphate; FAI–free androgen index ((testosterone/SHBG) × 100); FSH– follicle-stimulating hormone; LH–luteinizing hormone; SHBG–sex hormone binding globulin.
