# Supplementary material for: Polymorphism of the FSHB Gene Is Associated with Endometrial Hyperplasia
Source: Life (Basel). 2026 May 7;16(5):782. doi: 10.3390/life16050782 (PMC13208232; doi:10.3390/life16050782)
Supplement: Supplementary file 1 [file life-16-00782-s001.zip › Supplementary Table S3.pdf]

Supplementary Table S3. The allele and genotype frequencies of the studied SNPs in the endometrial hyperplasia and control groups.

Chr	SNP	Gene	Minor allele	Major allele	Minor allele frequency	Number of the studied chromosomes	Genotype distribution*	H_o	H_e	P_{HWE}
EH patients (n=520)										
7	rs148982377	*ZNF789*	C	T	0.057	1014	3/52/452	0.103	0.108	0.219
7	rs34670419	*ZKSCAN5*	T	G	0.038	1010	1/36/468	0.071	0.072	0.515
11	rs11031002	*FSHB*	A	T	0.073	992	2/68/426	0.137	0.135	1.000
11	rs11031005	*FSHB*	C	T	0.075	1010	1/74/430	0.147	0.139	0.345
11	rs112295236	*SLC22A10*	G	C	0.061	1006	0/61/442	0.121	0.114	0.244
12	rs117585797	*ANO2*	A	C	0.020	990	0/20/475	0.040	0.040	1.000
16	rs117145500	*CHD9*	C	A	0.102	992	8/85/403	0.171	0.183	0.213
17	rs727428	*SHBG*	T	C	0.376	998	62/251/186	0.503	0.469	0.127
17	rs1641549	*TP53*	T	C	0.224	1000	34/156/310	0.312	0.348	0.028
Control group (n=973)										
7	rs148982377	*ZNF789*	C	T	0.048	1888	1/88/855	0.093	0.091	0.718
7	rs34670419	*ZKSCAN5*	T	G	0.039	1890	2/70/873	0.074	0.075	0.650
11	rs11031002	*FSHB*	A	T	0.135	1862	17/218/696	0.234	0.234	1.000
11	rs11031005	*FSHB*	C	T	0.136	1894	20/217/710	0.229	0.235	0.488
11	rs112295236	*SLC22A10*	G	C	0.050	1874	2/90/845	0.096	0.095	1.000
12	rs117585797	*ANO2*	A	C	0.026	1866	1/46/886	0.049	0.050	0.462
16	rs117145500	*CHD9*	C	A	0.097	1862	7/167/757	0.179	0.175	0.707
17	rs727428	*SHBG*	T	C	0.385	1882	132/460/349	0.489	0.473	0.335
17	rs1641549	*TP53*	T	C	0.241	1860	61/326/543	0.350	0.366	0.209

Note: * minor allele homozygotes / heterozygotes / major allele homozygotes
