# Supplementary material for: Polymorphism of the FSHB Gene Is Associated with Endometrial Hyperplasia
Source: Life (Basel). 2026 May 7;16(5):782. doi: 10.3390/life16050782 (PMC13208232; doi:10.3390/life16050782)
Supplement: Supplementary file 1 [file life-16-00782-s001.zip › Supplementary Table S4.pdf]

Supplementary Table S4. Genotype combinations associated with EH.*

Model №	Combination №	Genotype combinations	*beta*	P	Risk, High/Low
		Two-order interaction models			
1	1	rs11031002 TT x rs11031005 TT	**1.03**	**3×10^{-8}**	**H**
	2	rs11031002 TA x rs11031005 TT	**-3.00**	**2×10^{-9}**	**L**
	3	rs11031002 TT x rs11031005 TC	-1.26	0.003	L
	4	rs11031002 TA x rs11031005 TC	-0.34	0.043	L
2	5	rs11031002 TT x rs112295236 CC	0.47	0.0005	H
	6	rs11031002 TA x rs112295236 CC	-0.84	0.000001	L
3	7	rs117145500 AA x rs11031002 TT	0.57	0.00001	H
	8	rs117145500 AA x rs11031002 TA	-0.87	0.000005	L
4	9	rs11031002 TT x rs148982377 TT	0.45	0.0009	H
	10	rs11031002 TA x rs148982377 TT	-0.73	0.00002	L
	11	rs11031002 TA x rs148982377 TC	-1.37	0.024	L
5	12	rs11031002 TT x rs117585797 CC	0.74	7×10^{-7}	H
	13	rs11031002 TA x rs117585797 CC	-0.83	0.000001	L
		Three-order interaction models			
1	1	rs11031002 TT x rs1641549 CC x rs11031005 TT	0.52	0.00001	H
	2	rs11031002 TA x rs1641549 CC x rs11031005 TT	-2.48	0.0002	L
	3	rs11031002 TA x rs1641549 CT x rs11031005 TT	-3.82	0.0003	L
	4	rs11031002 TA x rs1641549 TT x rs11031005 TT	-2.76	0.024	L
	5	rs11031002 TA x rs1641549 CC x rs11031005 TC	-0.61	0.006	L
	6	rs11031002 TT x rs1641549 CT x rs11031005 TC	-1.86	0.029	L
2	7	rs11031002 TT x rs117585797 CC x rs11031005 TT	**0.92**	**2×10^{-10}**	**H**
	8	rs11031002 TA x rs117585797 CC x rs11031005 TT	**-3.26**	**3×10^{-9}**	**L**
	9	rs11031002 TT x rs117585797 CC x rs11031005 TC	-1.40	0.001	L
3	10	rs11031002 TT x rs112295236 CC x rs11031005 TT	0.66	7×10^{-7}	H
	11	rs11031002 TA x rs112295236 CC x rs11031005 TT	**-3.56**	**9×10^{-9}**	**L**
	12	rs11031002 TT x rs112295236 CC x rs11031005 TC	-1.37	0.002	L
4	13	rs11031002 TT x rs727428 CC x rs11031005 TT	0.33	0.011	H
	14	rs11031002 TA x rs727428 CC x rs11031005 TT	-1.75	0.013	L
	15	rs11031002 TT x rs727428 CT x rs11031005 TT	0.36	0.002	H
	16	rs11031002 TA x rs727428 CT x rs11031005 TT	-3.55	0.000003	L
	17	rs11031002 TT x rs727428 CC x rs11031005 TC	-2.16	0.002	L
5	18	rs11031002 TT x rs11031005 TT x rs148982377 TT	0.63	0.000002	H
	19	rs11031002 TA x rs11031005 TT x rs148982377 TT	**-2.84**	**2×10^{-8}**	**L**
	20	rs11031002 TT x rs11031005 TC x rs148982377 TT	-1.51	0.002	L
	21	rs11031002 TT x rs11031005 TT x rs148982377 TC	0.46	0.033	H
		Four-order interaction models			
1	1	rs11031002 TT x rs1641549 CC x rs11031005 TT x rs34670419 GG	0.53	0.000009	H
	2	rs11031002 TA x rs1641549 CC x rs11031005 TT x rs34670419 GG	-2.36	0.0004	L
	3	rs11031002 TA x rs1641549 CT x rs11031005 TT x	-3.82	0.0003	L

		rs34670419 GG			
	4	rs11031002 TT x rs1641549 CC x rs11031005 TC x rs34670419 GG	-1.21	0.034	L
	5	rs11031002 TA x rs1641549 CC x rs11031005 TC x rs34670419 GG	-0.62	0.007	L
	6	rs11031002 TT x rs1641549 CT x rs11031005 TC x rs34670419 GG	-1.84	0.032	L
2	7	rs11031002 TT x rs117585797 CC x rs112295236 CC x rs11031005 TT	0.64	7×10^{-7}	H
	8	rs11031002 TA x rs117585797 CC x rs112295236 CC x rs11031005 TT	**-3.56**	**9×10^{-9}**	**L**
	9	rs11031002 TT x rs117585797 CC x rs112295236 CC x rs11031005 TC	-1.53	0.0009	L
3	10	rs11031002 TT x rs117585797 CC x rs727428 CC x rs11031005 TT	0.35	0.007	H
	11	rs11031002 TT x rs117585797 CC x rs727428 CC x rs11031005 TT	-2.23	0.006	L
	12	rs11031002 TT x rs117585797 CC x rs727428 CC x rs11031005 TT	0.38	0.002	H
	13	rs11031002 TT x rs117585797 CC x rs727428 CC x rs11031005 TT	-3.56	0.000002	H
	14	rs11031002 TT x rs117585797 CC x rs727428 CC x rs11031005 TC	-2.72	0.0008	L
4	15	rs11031002 TT x rs117585797 CC x rs11031005 TT x rs148982377 TT	0.63	0.000001	H
	16	rs11031002 TA x rs117585797 CC x rs11031005 TT x rs148982377 TT	**-3.10**	**2×10^{-8}**	**L**
	17	rs11031002 TT x rs117585797 CC x rs11031005 TC x rs148982377 TT	-1.74	0.0007	L
5	18	rs11031002 TT x rs112295236 CC x rs727428 CC x rs11031005 TT	0.40	0.028	H
	19	rs11031002 TA x rs112295236 CC x rs727428 CC x rs11031005 TT	-3.04	0.005	L
	20	rs11031002 TA x rs112295236 CC x rs727428 CT x rs11031005 TT	-3.52	0.000003	L
	21	rs11031002 TT x rs112295236 CG x rs727428 CT x rs11031005 TT	0.82	0.004	H
	22	rs11031002 TT x rs112295236 CC x rs727428 CC x rs11031005 TC	-2.12	0.003	L
6	23	rs11031002 TT x rs112295236 CC x rs11031005 TT x rs148982377 TT	0.42	0.0006	H
	24	rs11031002 TA x rs112295236 CC x rs11031005 TT x rs148982377 TT	**-3.41**	**5×10^{-8}**	**L**
	25	rs11031002 TT x rs112295236 CG x rs11031005 TT x rs148982377 TT	0.47	0.034	H
	26	rs11031002 TT x rs112295236 CC x rs11031005 TC x rs148982377 TT	-1.48	0.003	L
	27	rs11031002 TT x rs112295236 CC x rs11031005 TT x rs148982377 TC	0.56	0.017	H

Note: * Genotype combinations are derived from the interaction models obtained by the MB-MDR method and described in Table 4.
